# Global Dynamics of Avian Influenza Epidemic Models with Psychological Effect

**DOI:** 10.1155/2015/913726

**Published:** 2015-03-12

**Authors:** Sanhong Liu, Liuyong Pang, Shigui Ruan, Xinan Zhang

**Affiliations:** ^1^School of Mathematics and Statistics, Central China Normal University, Wuhan 430079, China; ^2^Department of Mathematics, Huanghuai University, Zhumadian 463000, China; ^3^Department of Mathematics, University of Miami, Coral Gables, FL 33124-4250, USA

## Abstract

Cross-sectional surveys conducted in Thailand and China after the outbreaks of the avian influenza A H5N1 and H7N9 viruses show a high degree of awareness of human avian influenza in both urban and rural populations, a higher level of proper hygienic practice among urban residents, and in particular a dramatically reduced number of visits to live markets in urban population after the influenza A H7N9 outbreak in China in 2013. In this paper, taking into account the psychological effect toward avian influenza in the human population, a bird-to-human transmission model in which the avian population exhibits saturation effect is constructed. The dynamical behavior of the model is studied by using the basic reproduction number. The results demonstrate that the saturation effect within avian population and the psychological effect in human population cannot change the stability of equilibria but can affect the number of infected humans if the disease is prevalent. Numerical simulations are given to support the theoretical results and sensitivity analyses of the basic reproduction number in terms of model parameters that are performed to seek for effective control measures for avian influenza.

## 1. Introduction

There are three types of influenza viruses: A, B, and C. Influenza A viruses are divided into subtypes based on two proteins on the surface of the virus: the hemagglutinin (H) and the neuraminidase (N) (CDC [1]). Avian influenza viruses with all 16 haemagglutinin [H1–H16] and all 9 neuraminidase [N1–N9] influenza A subtypes in the majority of possible combinations have been isolated from avian species (Alexander [2]). These viruses occur naturally among wild aquatic birds worldwide and can infect domestic poultry and other bird and animal species. Avian flu viruses do not normally infect humans. However, sporadic human infections with avian flu viruses have occurred (CDC [3]).

Avian influenza A viruses are classified into two categories: low pathogenic avian influenza A (LPAI) and highly pathogenic avian influenza A (HPAI). Three prominent subtypes of avian influenza A viruses known to infect both birds and people are influenza A H9, influenza A H5, and influenza A H7. All H9 viruses identified worldwide in wild birds and poultry are LPAI viruses. Rare, sporadic H9N2 virus infections of humans have been reported to cause generally mild upper respiratory tract illness. Most H5 and H7 viruses identified worldwide in wild birds and poultry are LPAI viruses. HPAI H5N1 virus infections in humans have been reported from 15 countries, often resulting in severe pneumonia with approximately 60 percent of mortality worldwide. H7 virus infection in humans is uncommon, but it has been documented in persons who have direct contact with infected birds, especially during outbreaks of H7 virus among poultry. In humans, LPAI (H7N2, H7N3, and H7N7) virus infections have caused mild to moderate illness; HPAI (H7N3, H7N7) virus infections have caused mild to severe and fatal illness. In March 2013, the first known human cases of infection with avian influenza H7N9 viruses were reported. They were associated with severe respiratory illness and death (CDC [4]).

In order to understand infectious diseases, mathematical models have been expensively used to analyze the epidemiological characteristics and then to provide useful control and prevention measures (Anderson and May [5], Keeling and Rohani [6]). In 2007, Iwami et al. [7] proposed ordinary differential equation (ODE) models to characterize the dynamical behavior of avian influenza between human and avian populations. Since then, various models have been developed to investigate different aspects of avian influenza transmitted by the H5N1 virus (see Lucchetti et al. [8], Iwami et al. [9], Jung et al. [10], Iwami et al. [11], Gumel [12], Agusto [13], Ma and Wang [14], Bourouiba et al. [15], Gourley et al. [16], Tuncer and Martcheva [17], etc.). Most recently, mathematical models have also been proposed to study the transmission of the avian influenza A H7N9 virus; see Zhang et al. [18], Xiao et al. [19], and Hsieh et al. [20].

Human behavior and social response play a very important role in the transmission of infectious diseases (Bauch and Galvani [21], Ferguson [22], and Funk et al. [23]). Understanding the impacts of human behavior and social response on the spread of infectious diseases are the key to improve control efforts (Funk et al. [24]). Toward the avian influenza, the general public reacts behaviourally, psychologically, and socially. After the outbreaks of the avian influenza A H5N1 virus, to determine the knowledge, attitudes, and practices relating to avian influenza in the general populations, cross-sectional surveys conducted in Thailand (Olsen et al. [25]) and China (Xiang et al. [26]) show a high degree of awareness of human avian influenza in both urban and rural populations and a higher level of proper hygienic practice among urban residents. To investigate human exposure to live-poultry and changes in risk perception and behavior after the March–May 2013 influenza A H7N9 outbreak in China, Wang et al. [27] surveyed 2,504 urban residents in 5 cities and 1,227 rural residents in 4 provinces and found that most (77%) urban respondents reported that they visited live markets less often after influenza A H7N9 outbreak. These certainly helped in controlling the further spread of avian influenza and and provide scientific support to assist the governments in developing strategies and health-education campaigns to prevent avian influenza infection among the general population.

Though behavioral and social responses to the spread of infectious diseases have been reported frequently, there is very little systematic study on their effect on the spread of infectious diseases (Funk et al. [24], Liu et al. [28]). Incorporating the dynamics of human behavior and social response in infectious diseases faces various challenges (Funk et al. [29]), for example, how to incorporate human behavior and social response in models of infectious disease dynamics and how to parameterize and measure the relevant human behavior and social response in such models.

It is well known that the incidence rate is an important factor in the transmission of infectious disease. In almost all recent models related to avian influenza, the incidence rate between susceptible avians and infective avians (and between susceptible humans and infective avians) takes the mass-action form with bilinear interactions, which is increasing and unbounded. As the surveys by Olsen et al. [25] and Xiang et al. [26] indicate, when more and more infected cases are reported via media, the general population would reduce their chances to visit the markets, so the incidence rate will in fact decrease. The nonmonotone incidence function (increasing at first, then decreasing when the number of infectives reaches a critical value, and bounding) proposed by Liu et al. [30] is suitable to model such a phenomenon; see also Ruan and Wang [31], Tang et al. [32], and Xiao and Ruan [33]. Similarly, the poultry farmers would take extra cautions and protection measures when the number of infective birds becomes larger and larger so that the incidence rate from infective birds to susceptible birds may saturate. This can be described by a saturated incidence function (increasing and bounding) used in Capasso and Serio [34].

In this paper, we construct an avian-human epidemic model with a nonlinear incidence rate for the spread of avian influenza virus from infective birds to susceptible birds describing the saturation effect within avian population and a nonmonotone incidence rate for the spread of avian influenza virus from infective birds to susceptible humans characterizing the psychological effect within humans. The paper is organized as follows. We describe the epidemic model and analyze the existence of the equilibria in Section 2. In Section 3, we study the global property of avian-only subsystem. The complete system is analyzed in Section 4. In Section 5, we present some numerical simulations to illustrate the theoretical results. A brief discussion is given in the last section.

## 2. The Avian-Human Influenza Epidemic Model and Equilibria

In our paper, we always assume that the avian influenza virus does not spread from person to person and mutate, and the domestic birds are the main infection source. The avian population is classified into two subclasses: susceptible and infective, denoted by *S*_*a*_(*t*) and *I*_*a*_(*t*), respectively, and the human population is classified into three subclasses: susceptible, infective, and recovered, denoted by *S*_*h*_(*t*), *I*_*h*_(*t*), and *R*_*h*_(*t*), respectively. In order to construct the corresponding model, we make the following assumptions.

(1) All new recruitments and newborns of the avian population and the human population are susceptible; the rate is denoted by Π_*a*_ and Π_*h*_, respectively.

(2) The avian influenza virus is contagious from an infective bird to a susceptible bird and from an infected bird to a susceptible human.

(3) When the number of the infective avians becomes larger, the incidence rate between infective avians and susceptible avians tends to a saturation level due to the protection measures taken by the poultry farmers or the crowding of the infected birds, which can be expressed as follows (Capasso and Serio [34]):

(1) $$\begin{array}{c} \frac{\beta_{a}S_{a}I_{a}}{1+bI_{a}}, \end{array}$$

where *β*_*a*_ is the transmission coefficient so that *β*_*a*_ *I*_*a*_ measures the infection force of the infective birds and *b* is the parameter which measures the inhibitory effort so that 1/(1 + *bI*_*a*_) describes the saturation due to the protection measures of the poultry farmers or the crowding of infected birds when the number of infective birds increases. Similarly, as the number of the infective human individuals increases, the susceptible human population may tend to reduce the number of contacts with infective avian population per unit time due to the psychological effect, so we use a nonmonotone incidence function (Liu et al. [30], Xiao and Ruan [33]) to describe the transmission of the virus from infective birds to susceptible humans; that is,

(2) $$\begin{array}{c} \frac{\beta_{h}S_{h}I_{a}}{1+cI_{h}^{2}}, \end{array}$$

where *β*_*h*_ *I*_*a*_ measures the infection force of the disease and 1/(1 + *cI*_*h*_^2^) describes the psychological effect from the behavioral change of the susceptible humans when the number of infective individuals becomes larger.

(4) An infected avian remains in the state of disease and cannot recover, but an infected human may recover and a recovered human has permanent immunity.

Based on the above assumptions, we have the following SI-SIR avian influenza model:

(3) dSadt=Πa−μaSa−βaIaSa1+bIa,dIadt=βaIaSa1+bIa−μa+δaIa,dShdt=Πh−μhSh−βhIaSh1+cIh2,dIhdt=βhIaSh1+cIh2−μh+δh+γIh,dRhdt=γIh−μhRh,

where *μ*_*a*_ (*μ*_*h*_) is the natural death rate of the avian population (the human population) respectively; *δ*_*a*_ (*δ*_*h*_) is the disease-related death rate of the infected avian (the infected human), respectively; *γ* is the natural recovery rate of the infective human. We assume that *b*, *c* are nonnegative constants and all the other parameters are positive.

Notice that the model proposed in Iwami et al. [7] is a special case of model (3) with being *b* = 0 and *c* = 0 if we ignore the virus mutation and spreading from human to human.

In the following, we will analyze the global dynamics of the above system. We first analyze the existence of the equilibria. It is clear that system (3) has a unique solution satisfying the initial conditions in ℝ_+_^5^ which is a positively invariant set. The equilibrium (*S*_*a*_, *I*_*a*_, *S*_*h*_, *I*_*h*_, *R*_*h*_) of system (3) must satisfy the following equations:

(4) $$\begin{array}{c} \Pi_{a}-\mu_{a}S_{a}-\frac{\beta_{a}I_{a}S_{a}}{1+bI_{a}}=0,\\ \frac{\beta_{a}I_{a}S_{a}}{1+bI_{a}}-\left(\mu_{a}+\delta_{a}\right)I_{a}=0,\\ \Pi_{h}-\mu_{h}S_{h}-\frac{\beta_{h}I_{a}S_{h}}{1+cI_{h}^{2}}=0,\\ \frac{\beta_{h}I_{a}S_{h}}{1+cI_{h}^{2}}-\left(\mu_{h}+\delta_{h}+\gamma \right)I_{h}=0,\\ \gamma I_{h}-\mu_{h}R_{h}=0. \end{array}$$

We consider two cases.

**Case 1 .**

If *I*_*a*_ = 0, then *S*_*a*_ = Π_*a*_/*μ*_*a*_, *S*_*h*_ = Π_*h*_/*μ*_*h*_, *I*_*h*_ = 0, and *R*_*h*_ = 0, which shows that system (3) always has a unique disease-free equilibrium given by (*S*_a_^*^, 0, *S*_h_^*^, 0,0) with *S*_a_^*^ = Π_*a*_/*μ*_*a*_ and *S*_h_^*^ = Π_*h*_/*μ*_*h*_.

We adopt the notation and method of van den Driessche and Watmough [35] to calculate the basic reproduction number. According to [35], system (3) can be written as

(5) $$\begin{array}{c} \frac{dX}{dt}=F-V, \end{array}$$

where

(6) Xt=IatIhtSatShtRht,  F=βaIaSa1+bIaβhIaSh1+cIh2000,V=(μa+δa)Ia(μh+δh+γ)IhμaSa+βaIaSa1+bIa−ΠaμhSh+βhIaSh1+cIh2−ΠhμhRh−γIh.

The infective compartments are *I*_*a*_ and *I*_*h*_. Then,

(7) F=βaSa∗0βhSh∗0,  V=μa+δa00μh+δh+γ,FV−1=βaSa∗μa+δa0βhSh∗μh+δh+γ0.

Hence, following [35], we obtain the basic reproduction number

(8) $$\begin{array}{c} R_{0}=\frac{\beta_{a}S_{a}^{*}}{\mu_{a}+\delta_{a}}=\frac{\beta_{a}\Pi_{a}}{\mu_{a}\left(\mu_{a}+\delta_{a}\right)}. \end{array}$$

**Case 2 .**

If *I*_*a*_ ≠ 0, then *S*_*a*_ = (*μ*_*a*_ + *δ*_*a*_)(1 + *bI*_*a*_)/*β*_*a*_ from the second equation of (4). Substituting *S*_*a*_ for (*μ*_*a*_ + *δ*_*a*_)(1 + *bI*_*a*_)/*β*_*a*_ in the first equation of system (4), we obtain that

(9) $$\begin{array}{c} I_{a}=\frac{\mu_{a}\left(R_{0}-1\right)}{\mu_{a}b+\beta_{a}}≜I_{a}^{**}. \end{array}$$

*I*_a_^**^ > 0 when ℛ_0_ > 1. Hence, *S*_*h*_, *I*_*h*_, and *R*_*h*_ satisfy the following equations:

(10) $$\begin{array}{c} \Pi_{h}-\mu_{h}S_{h}-\frac{\beta_{h}I_{a}^{**}S_{h}}{1+cI_{h}^{2}}=0,\\ \frac{\beta_{h}I_{a}^{**}S_{h}}{1+cI_{h}^{2}}-\left(\mu_{h}+\delta_{h}+\gamma \right)I_{h}=0,\\ \gamma I_{h}-\mu_{h}R_{h}=0. \end{array}$$

From the equations in (10), we have *S*_*h*_ = (*μ*_*h*_ + *δ*_*h*_ + *γ*)*I*_*h*_(1 + *cI*_*h*_^2^)/*β*_*h*_ *I*_a_^**^, *R*_*h*_ = *γI*_*h*_/*μ*_*h*_, and *I*_*h*_ satisfies the following equation:

(11) gIh=μh(μh+δh+γ)cIh3+(μh+δh+γ)(μh+βhIa∗∗)Ih−ΠhβhIa∗∗=0.

Obviously, *g*(0) < 0, *g*(*I*_*h*_)→+*∞* as *I*_*h*_ → +*∞*, and *dg*/*dI*_*h*_ = 3(*μ*_*h*_ + *δ*_*h*_ + *γ*)*cI*_*h*_^2^ + (*μ*_*h*_ + *δ*_*h*_ + *γ*)(*μ*_*h*_ + *β*_*h*_ *I*_a_^**^) > 0 which indicates that *g*(*I*_*h*_) is strictly monotonically increasing. Hence, equation *g*(*I*_*h*_) = 0 has only one positive root *I*_*h*_ = *I*_h_^**^.

In summary, we have the following results.

**Lemma 1 .**

(i) If ℛ_0_ ≤ 1, system (3) has only a disease-free equilibrium given by *A*(*S*_a_^*^, 0, *S*_h_^*^, 0,0). (ii) If ℛ_0_ > 1, system (3) has two equilibria: a disease-free equilibrium *A* and an endemic equilibrium *B*(*S*_a_^**^, *I*_a_^**^, *S*_h_^**^, *I*_h_^**^, *R*_h_^**^), where *S*_a_^*^ = Π_*a*_/*μ*_*a*_, *S*_h_^*^ = Π_*h*_/*μ*_*h*_, *S*_a_^**^ = (*μ*_*a*_ + *δ*_*a*_)(1 + *bI*_a_^**^)/*β*_*a*_, *I*_a_^**^ = *μ*_*a*_(ℛ_0_ − 1)/(*μ*_*a*_ *b* + *β*_*a*_), *R*_h_^**^ = *γI*_h_^**^/*μ*_*h*_, *S*_h_^**^ = (*μ*_*h*_ + *δ*_*h*_ + *γ*)*I*_h_^**^(1 + *cI*_h_^**^^2^)/*β*_*h*_ *I*_a_^**^, and *I*_h_^**^ satisfies (11).

## 3. Analysis of the Avian-Only Submodel

Note that the first two equations are independent in system (3), so we study the dynamical behavior of the avian-only system firstly, which takes the following form:

(12) $$\begin{array}{c} \frac{dS_{a}}{dt}=\Pi_{a}-\mu_{a}S_{a}-\frac{\beta_{a}I_{a}S_{a}}{1+bI_{a}},\\ \frac{dI_{a}}{dt}=\frac{\beta_{a}I_{a}S_{a}}{1+bI_{a}}-\left(\mu_{a}+\delta_{a}\right)I_{a}. \end{array}$$

Based on Lemma 1, system (12) always has a disease-free equilibrium *A*_*a*_(*S*_a_^*^, 0) and a unique endemic equilibrium *B*_*a*_(*S*_a_^**^, *I*_a_^**^) if ℛ_0_ > 1, where the expressions of *S*_a_^*^, *S*_a_^**^, *I*_a_^**^, and ℛ_0_ have been given in Section 2. Note that ℛ_0_ is also the basic reproduction of system (12). We will discuss the properties of system (12) in the positively invariant set ℝ_+_^2^.

**Lemma 2 .**

(i) The disease-free equilibrium *A*_*a*_(*S*_a_^*^, 0) is locally asymptotically stable if ℛ_0_ ≤ 1. (ii) The endemic equilibrium *B*_*a*_(*S*_a_^**^, *I*_a_^**^) is locally asymptotically stable if ℛ_0_ > 1.

**Proof**

(i) The characteristic equation of the Jacobian matrix around the equilibrium *A*_*a*_ is

(13) $$\begin{array}{c} \left(\lambda +\mu_{a}\right)\left(\lambda +\left(\mu_{a}+\delta_{a}\right)\left(1-R_{0}\right)\right)=0. \end{array}$$

Clearly, the eigenvalues of the above characteristic equation are *λ*_1_ = −*μ*_*a*_ < 0, *λ*_2_ = −(*μ*_*a*_ + *δ*_*a*_)(1 − ℛ_0_). Hence, the equilibrium *A*_*a*_ is locally asymptotically stable if ℛ_0_ < 1 but unstable if ℛ_0_ > 1.

(ii) If ℛ_0_ > 1, system (12) has a unique positive equilibrium *B*_*a*_. Similarly, we can obtain the characteristic equation of the Jacobian matrix around the equilibrium *B*_*a*_ as follows:

(14) $$\begin{array}{c} \lambda^{2}+A\lambda +B=0, \end{array}$$

where

(15) A=μa+βaIa∗∗1+bIa∗∗+(μa+δa)−βaSa∗∗1+bIa∗∗2=μa+Ia∗∗1+bIa∗∗(βa+(μa+δa)b),B=μa(μa+δa)+(μa+δa)βaIa∗∗1+bIa∗∗−βaμaSa∗∗1+bIa∗∗2=(μa+δa)Ia∗∗1+bIa∗∗(βa+bμa).

Since *S*_a_^**^ > 0, *I*_a_^**^ > 0 if ℛ_0_ > 1, then *A* > 0, *B* > 0, so the two eigenvalues of characteristic equation *λ* ^2^ + *Aλ* + *B* = 0 have negative real parts. Hence, the positive equilibrium *B*_*a*_ is locally asymptotically stable.

**Lemma 3 .**

(i) The disease-free equilibrium *A*_*a*_(*S*_a_^*^, 0) is globally asymptotically stable if ℛ_0_ ≤ 1. (ii) The endemic equilibrium *B*_*a*_(*S*_a_^**^, *I*_a_^**^) is globally asymptotically stable if ℛ_0_ > 1.

**Proof**

(i) If ℛ_0_ ≤ 1, we can choose a Liapunov function

(16) $$\begin{array}{c} V=S_{a}-S_{a}^{*}-S_{a}^{*}\ln\frac{S_{a}}{S_{a}^{*}}+I_{a}. \end{array}$$

Then,

(17) dVdt(12)=Sa−Sa∗Sa(Πa−μaSa−βaIaSa1+bIa)+βaIaSa1+bIa−(μa+δa)Ia=−μaSaSa−Sa∗2−βaIa(Sa−Sa∗)1+bIa+βaSaIa1+bIa−(μa+δa)Ia=−μaSaSa−Sa∗2+βaSa∗Ia1+bIa−(μa+δa)Ia<−μaSaSa−Sa∗2+Ia(μa+δa)(R0−1)≤ 0.

Since *D* = {(*S*_*a*_, *I*_*a*_) ∈ ℝ_+_^2^ : *dV*/*dt* = 0} = {(*S*_*a*_, *I*_*a*_) ∈ ℝ_+_^2^ : *S*_*a*_ = *S*_a_^*^, *I*_*a*_ = 0} = {*A*_*a*_}, according to LaSalle's invariance principle [36], *A*_*a*_ is globally asymptotically stable.

(ii) In order to prove the global stability of the endemic equilibrium *B*_*a*_ in ℝ_+_^2^, we use Bendixson-Dulac criteria [37, 38]. For the sake of convenience, let

(18) $$\begin{array}{c} f_{1}=\Pi_{a}-\mu_{a}S_{a}-\frac{\beta_{a}I_{a}S_{a}}{1+bI_{a}},\\ g_{1}=\frac{\beta_{a}I_{a}S_{a}}{1+bI_{a}}-\left(\mu_{a}+\delta_{a}\right)I_{a}. \end{array}$$

In order to use the Bendixson-Dulac criteria, we choose a positive smooth function *B*_1_ = (1 + *bI*_*a*_)/*β*_*a*_ *I*_*a*_ *S*_*a*_. Then, *B*_1_, *f*_1_, and *g*_1_ are continuously differentiable functions in the region ℝ_+_^2^, and

(19) $$\begin{array}{c} \frac{\partial \left(B_{1}f_{1}\right)}{\partial S_{a}}+\frac{\partial \left(B_{1}g_{1}\right)}{\partial I_{a}}=-\frac{\Pi_{a}\left(1+bI_{a}\right)}{\beta_{a}I_{a}S_{a}^{2}}-\frac{b\left(\mu_{a}+\delta_{a}\right)}{\beta_{a}S_{a}}. \end{array}$$

It is obvious that the sign of the above expression is negative, which shows the nonexistence of any closed orbit. Therefore, *B*_*a*_ is globally asymptotically stable if ℛ_0_ > 1.

## 4. Analysis of the Avian-Human Influenza Epidemic Model

Since the first four equations of system (3) are independent of the variable *R*_*h*_, we only need to analyze the dynamical behavior of the following equivalent system:

(20) $$\begin{array}{c} \frac{dS_{a}}{dt}=\Pi_{a}-\mu_{a}S_{a}-\frac{\beta_{a}I_{a}S_{a}}{1+bI_{a}},\\ \frac{dI_{a}}{dt}=\frac{\beta_{a}I_{a}S_{a}}{1+bI_{a}}-\left(\mu_{a}+\delta_{a}\right)I_{a},\\ \frac{dS_{h}}{dt}=\Pi_{h}-\mu_{h}S_{h}-\frac{\beta_{h}S_{h}I_{a}}{1+cI_{h}^{2}},\\ \frac{dI_{h}}{dt}=\frac{\beta_{h}S_{h}I_{a}}{1+cI_{h}^{2}}-\left(\mu_{h}+\delta_{h}+\gamma \right)I_{h}. \end{array}$$

We will discuss the dynamical behavior of system (20) in the positive invariant set ℝ_+_^4^.

### 4.1. Local Stability

According to Lemma 1, system (20) always has a disease-free equilibrium given by *A*_*ah*_(*S*_a_^*^, 0, *S*_h_^*^, 0); if ℛ_0_ > 1, system (20) also has a unique endemic equilibrium *B*_*ah*_(*S*_a_^**^, *I*_a_^**^, *S*_h_^**^, *I*_h_^**^), where *S*_a_^*^, *S*_h_^*^, *S*_a_^**^, *I*_a_^**^, *S*_h_^**^, *I*_h_^**^, and ℛ_0_ have been given in Section 2. And ℛ_0_ is also the basic reproduction of system (20).

Similarly, we have the following properties.

**Lemma 4 .**

(i) The disease-free equilibrium *A*_*ah*_ is locally asymptotically stable for positive trajectories if ℛ_0_ ≤ 1 but unstable if ℛ_0_ > 1. (ii) The endemic equilibrium *B*_*ah*_ is locally asymptotically stable for positive trajectories if ℛ_0_ > 1.

**Proof**

The Jacobian matrix of system (20) around an arbitrary equilibrium (*S*_*a*_, *I*_*a*_, *S*_*h*_, *I*_*h*_) is

(21) J=−μa−βaIa1+bIa−βaSa1+bIa200βaIa1+bIaβaSa1+bIa2−(μa+δa)000−βhSh1+cIh2−μh−βhIa1+cIh22cβhIhShIa1+cIh220βhSh1+cIh2βhIa1+cIh2−2cβhIhShIa1+cIh22−(μh+δh+γ).

(i) If (*S*_*a*_, *I*_*a*_, *S*_*h*_, *I*_*h*_) = (*S*_a_^*^, 0, *S*_h_^*^, 0), the eigenvalues are *λ*_1_ = −*μ*_*a*_, *λ*_2_ = (*μ*_*a*_ + *δ*_*a*_)(ℛ_0_ − 1), *λ*_3_ = −*μ*_*h*_, and *λ*_4_ = −(*μ*_*h*_ + *δ*_*h*_ + *γ*).

If ℛ_0_ < 1, all the eigenvalues are negative; hence, the equilibrium *A*_*ah*_ is locally asymptotically stable. But if ℛ_0_ > 1, due to the eigenvalue *λ*_2_ > 0, then the equilibrium *A*_*ah*_ is unstable.

(ii) If ℛ_0_ > 1, system (20) exists a unique endemic equilibrium *B*_*ah*_. The characteristic equation of the Jacobian matrix at the endemic equilibrium *B*_*ah*_ is

(22) $$\begin{array}{c} \left(\lambda^{2}+A\lambda +B\right)\left(\lambda^{2}+C\lambda +D\right)=0, \end{array}$$

where *A* and *B* satisfy (15) and

(23) C=μh+βhIa∗∗1+cIh∗∗2+2cβhSh∗∗Ia∗∗Ih∗∗1+cIh∗∗22+μh+δh+γ,D=μh(μh+δh+γ)+2cμhβhSh∗∗Ia∗∗Ih∗∗1+cIh∗∗22+(μh+δh+γ)βhIa∗∗1+cIh∗∗2.

According to Lemma 2, the eigenvalues of equation *λ* ^2^ + *Aλ* + *B* = 0 have negative real parts, so the stability of equilibrium *B*_*ah*_ is decided by the eigenvalues of equation *λ* ^2^ + *Cλ* + *D* = 0.

Since *S*_h_^**^ > 0, *I*_h_^**^ > 0 when ℛ_0_ > 1, then *C* > 0, *D* > 0. Hence, both eigenvalues of equation *λ* ^2^ + *Cλ* + *D* = 0 have negative real parts. Therefore, the endemic equilibrium *B*_*ah*_ is locally asymptotically stable.

### 4.2. Global Stability

**Theorem 5 .**

(i) If ℛ_0_ ≤ 1, then the disease-free equilibrium *A*_*ah*_ of system (20) is globally asymptotically stable. (ii) If ℛ_0_ > 1, the endemic equilibrium *B*_*ah*_ of system (20) is globally asymptotically stable.

**Proof**

(i) According to Lemma 3, the disease-free equilibrium *A*_*a*_ of the avian-only system (12) is globally asymptotically stable if ℛ_0_ ≤ 1. To prove the global stability of *A*_*ah*_, we only need to consider system (20) with the avian components already at the disease-free steady state, given by

(24) dShdt=Πh−μhSh,dIhdt=−(μh+δh+γ)Ih.

Clearly, we can obtain that *S*_*h*_ → *S*_h_^*^, *I*_*h*_ → 0 if *t* → *∞*. Hence, the disease-free equilibrium *A*_*ah*_ is globally asymptotically stable if ℛ_0_ ≤ 1.

(ii) Similarly, by Lemma 3, the endemic equilibrium *B*_*a*_ of subsystem (12) is globally asymptotically stable in the ℝ_+_^2^ if ℛ_0_ > 1. To prove the global stability of the equilibrium *B*_*ah*_ in the region ℝ_+_^4^, we only need to consider system (20) with the avian components already at the endemic steady state, given by

(25) $$\begin{array}{c} \frac{dS_{h}}{dt}=\Pi_{h}-\frac{\beta_{h}S_{h}I_{a}^{**}}{1+cI_{h}^{2}}-\mu_{h}S_{h},\\ \frac{dI_{h}}{dt}=\frac{\beta_{h}S_{h}I_{a}^{**}}{1+cI_{h}^{2}}-\left(\mu_{h}+\delta_{h}+\gamma \right)I_{h}. \end{array}$$

We can easily deduce that system (25) has a unique positive equilibrium (*S*_h_^**^, *I*_h_^**^) which is locally asymptotically stable. To prove the global stability of the equilibrium (*S*_h_^**^, *I*_h_^**^) in the region ℝ_+_^2^, we adopt the Bendixson-Dulac criteria [37, 38].

For convenience, we note that

(26) $$\begin{array}{c} f_{2}=\Pi_{h}-\frac{\beta_{h}S_{h}I_{a}^{**}}{1+cI_{h}^{2}}-\mu_{h}S_{h},\\ g_{2}=\frac{\beta_{h}S_{h}I_{a}^{**}}{1+cI_{h}^{2}}-\left(\mu_{h}+\delta_{h}+\gamma \right)I_{h}. \end{array}$$

In order to use the Bendixson-Dulac criteria, we choose a positive smooth function *B*_2_ = (1 + *cI*_*h*_^2^)/*β*_*h*_ *S*_*h*_ *I*_a_^**^ and then *B*_2_, *f*_2_, and *g*_2_ are continuously differentiable functions in the region ℝ_+_^2^ and

(27) ∂(B2f2)∂Sh+∂(B2g2)∂Ih  =−Πh(1+cIh2)βhIa∗∗Sh2−(1+3cIh2)(μh+δh+γ)βhShIa∗∗<0.

According to the Bendixson-Dulac Theorem [37, 38], there is no closed orbit in the region ℝ_+_^2^. Therefore, (*S*_h_^**^, *I*_h_^**^) is globally asymptotically stable. Hence, the endemic equilibrium *B*_*ah*_ is globally asymptotically stable if ℛ_0_ > 1.

**Corollary 6 .**

(i) The disease-free equilibrium *A* of system (3) is globally asymptotically stable for positive trajectories if ℛ_0_ ≤ 1 but unstable if ℛ_0_ > 1. (ii) The unique endemic equilibrium *B* of system (3) is globally asymptotically stable if ℛ_0_ > 1.

**Remark 7 .**

The basic reproduction number ℛ_0_ is a threshold value which determines whether the avian influenza disappears or not and parameter *β*_*a*_, the transmission rate from infective birds to susceptible birds, is a key parameter which influences the basic reproduction number ℛ_0_.

**Remark 8 .**

Although our results are similar to those in Iwami et al. [7] when the virus mutation and spreading from human to human are not considered, parameters *b* and *c* influence the number of infected humans dramatically. We will show this point in the next section via numerical simulations.

## 5. Numerical Simulations and Sensitivity Analysis

In this section, for the sake of convenience and simplicity, we always keep some parameters fixed as follows: Π_*a*_ = 350, *μ*_*a*_ = 0.01, *δ*_*a*_ = 0.05, Π_*h*_ = 100, *μ*_*h*_ = 3.91∗10^−3^, *δ*_*h*_ = 0.3, and *γ* = 0.01. Generally speaking, avian influenza mainly outbreaks in a specific location. We estimate that the number of susceptible avian population is between 100000 and 1000000 and the number of infective avian population is between 0 and 100 every day in the region. Then, we choose initial values as (*S*_*a*_(0), *I*_*a*_(0), *S*_*h*_(0), *I*_*h*_(0), *R*_*h*_(0)) = (100000, 100, 100000, 1, 0).

### 5.1. Numerical Simulations

It should be noted that the transmission rate from infective birds to susceptible birds *β*_*a*_ is a key parameter which influences the size of the basic reproduction number ℛ_0_, the parameter *b* describes the saturation effect within the avian population, and *c* represents the psychological effect of the general public toward the infective individuals within the human population. Moreover, *β*_*h*_ is also an important parameter which influences the number of infective human individuals. In this subsection, we investigate the influence of parameters *β*_*a*_, *β*_*h*_, *b*, and *c* on the number of infected humans by performing some numerical simulations.

**Figure 1:**
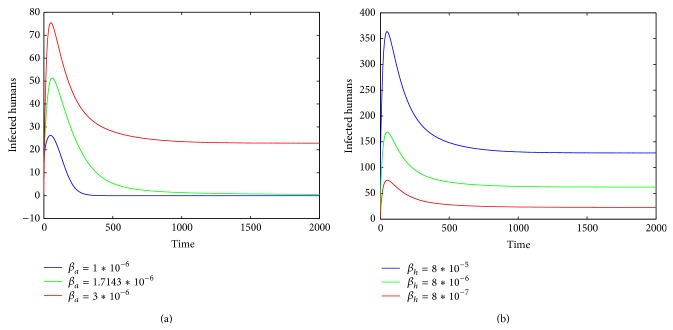
The plots display the changes of *I*_*h*_(*t*) in terms of *β*_*a*_ or *β*_*h*_, where parameters *b*, *c* are fixed as *b* = *c* = 0.001. (a) *β*_*a*_ varies and *β*_*h*_ = 8∗10^−7^ and (b) *β*_*h*_ varies and *β*_*a*_ = 3∗10^−6^.

Firstly, we study the influence of parameters *β*_*a*_, *β*_*h*_ on the number of infective individuals. When parameters Π_*a*_, *μ*_*a*_, and *δ*_*a*_ are fixed as the above, the threshold value *β*_a_^*^ = 1.7143∗10^−6^, so that ℛ_0_ = 1. If *β*_*a*_ ≤ *β*_a_^*^, the disease will die out, and the solution *I*_*h*_(*t*) is asymptotically stable and converges to the disease-free state value. If *β*_*a*_ > *β*_a_^*^, the disease is prevalent and the solution *I*_*h*_(*t*) is asymptotically stable and converges to the endemic state value (see Figure 1(a)). When we fix *β*_*a*_, for instance, *β*_*a*_ = 3∗10^−6^, then ℛ_0_ > 1. We can see that the solution *I*_*h*_(*t*) is approaching the endemic state value and increases with the increasing of *β*_*h*_ (see Figure 1(b)).

Secondly, we investigate the influence of parameters *b* and *c* on the number of infected humans. If parameter *β*_*a*_ = 3∗10^−6^, then ℛ_0_ = 1.75 > 1, the disease is endemic, and the solution *I*_*h*_(*t*) is asymptotically stable and converges to the endemic state value.

**Figure 2:**
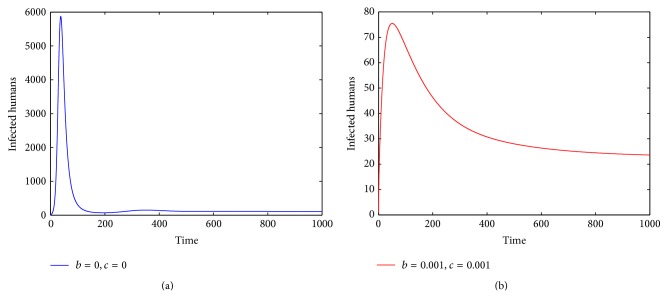
The plots present the changes of *I*_*h*_(*t*) (a) without or (b) with the saturation effect and psychological effect when ℛ_0_ > 1. The solution *I*_*h*_(*t*) is asymptotically stable and converges to the endemic state value.

**Figure 3:**
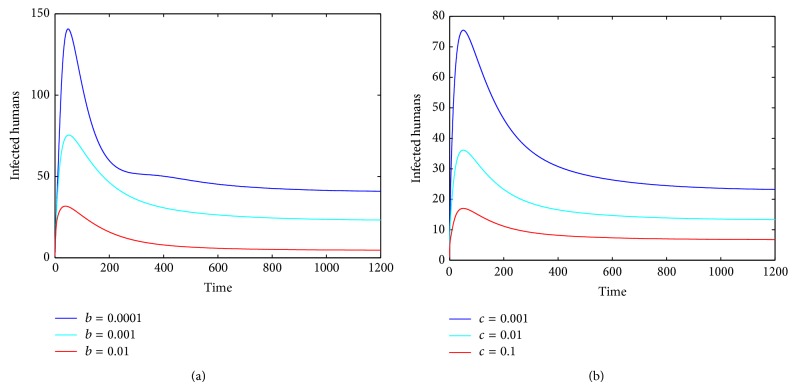
The plots reveal that different values of the other parameter influence the values of *I*_*h*_(*t*) when one parameter is fixed and ℛ_0_ > 1. (a) Parameter *b* influences the values of *I*_*h*_(*t*) with *c* = 0.001; (b) parameter *c* influences the values of *I*_*h*_(*t*) with *b* = 0.001.

For comparison, when parameters *b*, *c* are chosen as *b* = 0, *c* = 0 and *b* = 0.001, *c* = 0.001, respectively, we observe that subtle changes on parameters decrease the number of infected humans dramatically (see Figure 2). When parameter *c* is fixed and the other parameter *b* takes different values, we can see that the number of infected humans decreases obviously when parameter *b* increases (see Figure 3(a)). When parameter *b* is fixed and parameter *c* takes different values, we have a similar result (see Figure 3(b)).

If parameter *β*_*a*_ = 1∗10^−6^, then ℛ_0_ = 0.5833 < 1, the disease disappears, and the solution *I*_*h*_(*t*) is asymptotically stable and converges to the disease-free state value.

**Figure 4:**
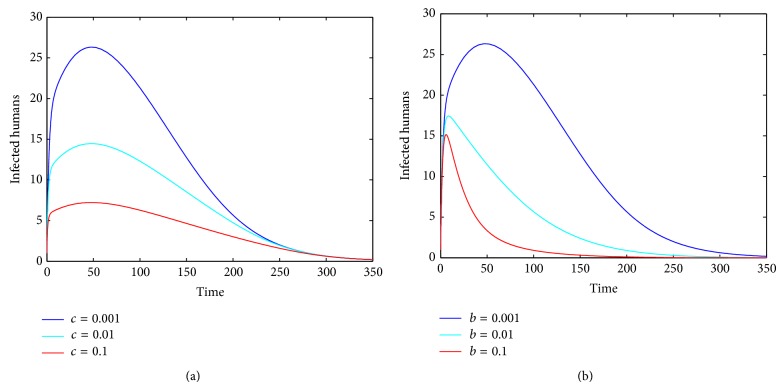
The plots indicate that different values of the other parameter influence the values of *I*_*h*_(*t*) when one parameter is fixed and ℛ_0_ < 1. (a) Parameter *c* influences the values of *I*_*h*_(*t*) with *b* = 0.001; (b) parameter *b* influences the values of *I*_*h*_(*t*) with *c* = 0.001.

**Figure 5:**
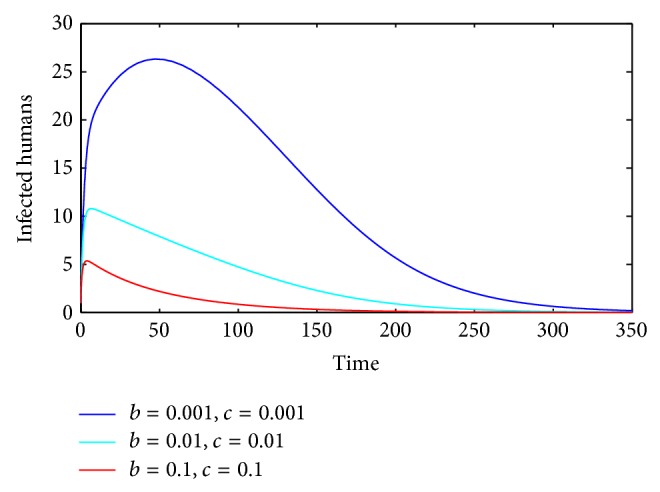
The plots show that different values of both parameters *b* and *c* influence the values of *I*_*h*_(*t*) when ℛ_0_ < 1. The solution *I*_*h*_(*t*) is asymptotically stable and converges to the disease-free state value.

Similarly, when parameter *b* is fixed, we can observe that the number of infected humans decreases with the increase of parameter *c* (see Figure 4(a)). When we fix parameter *c* and let parameter *b* change, we obtain a similar result (see Figure 4(b)). When both parameters *b* and *c* change simultaneously, we can see that the number of infected humans decreases obviously with the increase of parameters *b* and *c* (see Figure 5).

### 5.2. Sensitivity Analysis

**Figure 6:**
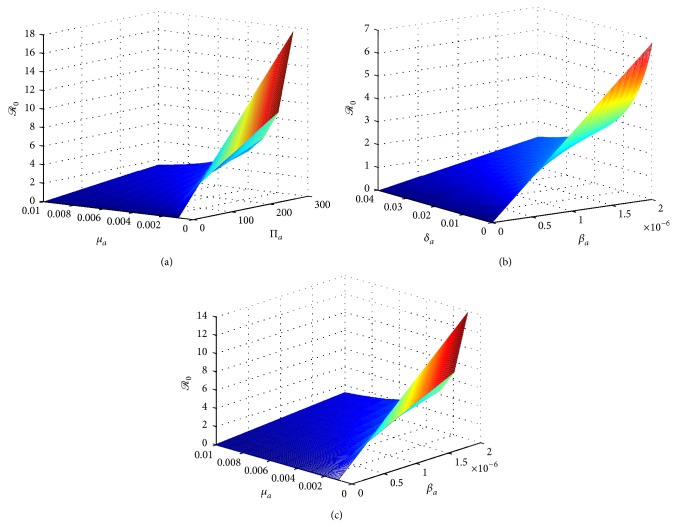
Plots of the basic reproduction number ℛ_0_ on other parameters: (a) in terms of parameters Π_*a*_ and *μ*_*a*_; (b) in terms of parameters *β*_*a*_ and *δ*_*a*_; (c) in terms of parameters *β*_*a*_ and *μ*_*a*_.

In order to find better control strategies for avian influenza infections, we firstly investigate which parameters can reduce the basic reproduction number ℛ_0_. From Figure 6, we can observe that ℛ_0_ decreases if *β*_*a*_ or Π_*a*_ decreases or if parameters *μ*_*a*_ or *δ*_*a*_ increase. If the contact rate *β*_*a*_ is sufficiently small, then avian influenza could be eliminated even if *μ*_*a*_ = 0 or *δ*_*a*_ = 0. But it is difficult to control *β*_*a*_. *δ*_*a*_ is hard to control too because the disease-related death rate is a relative fixed constant. Even if *β*_*a*_ is not small, ℛ_0_ could be less than 1 as long as *μ*_*a*_ is large enough and Π_*a*_ is small enough. It is feasible if we shorten the lifetime of domestic birds or reduce all new recruitments and newborns of domestic birds. The optimal control strategy will be a combination of shortening lifetime of domestic birds and reducing all new recruitments and newborns of domestic birds.

**Figure 7:**
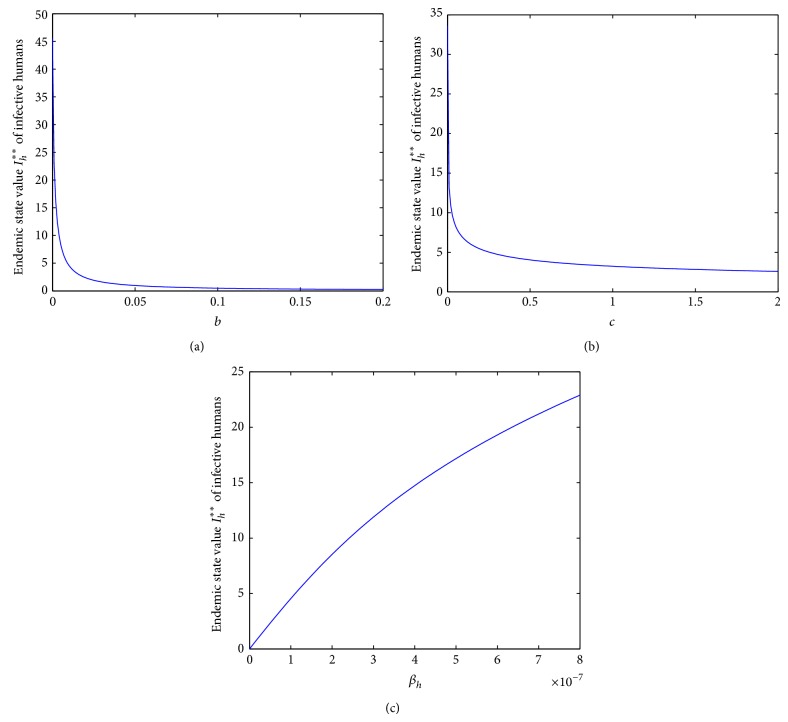
The plots show the influence of parameters *b*, *c*, and *β*_*h*_ on the endemic state value of infective human individuals *I*_h_^**^ with *β*_*a*_ = 3∗10^−6^. (a) Parameter *b* influences the values of *I*_h_^**^ with *c* = 0.001, *β*_*h*_ = 8∗10^−7^; (b) parameter *c* influences the values of *I*_h_^**^ with *b* = 0.001, *β*_*h*_ = 8∗10^−7^; (c) parameter *β*_*h*_ influences the values of *I*_h_^**^ with *b* = *c* = 0.001.

Secondly, we investigate the influence of parameters *b*, *c*, and *β*_*h*_ on the endemic state value of the infective human individuals *I*_h_^**^. We can observe that *I*_h_^**^ decreases with the increase of parameters *b* or *c* and will tend to 0 when parameters *b* or *c* increase to infinity from Figures 7(a) and 7(b). We can also observe that *I*_h_^**^ increases with the increase of parameter *β*_*h*_ from Figure 7(c). It suggests that we can increase parameters *b* and *c* or reduce parameter *β*_*h*_ to control the disease to a lower level. Similarly, it is difficult to control parameter *b*. However, we can control parameters *c* and *β*_*h*_. For example, we can enhance the intensity of media coverage to enhance the psychological effect of the human population; we can reduce *β*_*h*_ through susceptible human individuals by avoiding as far as possible the contact with the infective avian population. From Figure 7(c), we can also see that if *β*_*h*_ = 0, avian influenza will be eliminated which implies that closing the retail live-poultry markets is an effective control measure.

## 6. Discussion

In this paper, to study the transmission dynamics of avian influenza from birds to humans, we constructed a nonlinear ordinary differential equation model considering the saturation effect within the avian population and the psychological effect of the general public toward the outbreaks of avian influenza. We obtained a threshold value for the prevalence of avian influenza and discussed the local and global asymptotical stability of each equilibrium of the nonlinear system. Our results indicate that the asymptotic dynamics of the model are completely determined by the threshold value ℛ_0_: the disease-free equilibrium exists and is globally asymptotically stable if ℛ_0_ ≤ 1; the disease-free equilibrium becomes unstable and the endemic equilibrium exists and is globally asymptotically stable if ℛ_0_ > 1. In other words, the avian influenza disappears if ℛ_0_ ≤ 1 but is prevalent and becomes endemic if ℛ_0_ > 1. Our numerical simulations (see Figures 1–5) support the theoretical results.

Our results demonstrate that transmission dynamics of the avian influenza are greatly determined by the infection force of the disease in birds. It should be noted that the saturation effect within the avian population *b*, the psychological effect within the human population *c*, and the transmission coefficient from infective birds to susceptible humans *β*_*h*_ do not affect the stability of the equilibria and thus the transmission and outbreaks of the disease, since infected humans do not spread the virus any further. However, the theoretical analyses and numerical simulations indicate that increasing parameters *b* and *c* and the reducing parameter *β*_*h*_ could reduce the number of the infected humans and may help to control the disease.

Numerical simulation suggests that the optimal control strategy should be a combination of shortening the lifetime of domestic birds, reducing all new recruitments and newborns of domestic birds, and reducing contacts between susceptible birds and infective birds. If there is an outbreak of avian influenza, the effect control measures should be a combination of enhancing the intensity of media coverage and avoiding contact with the infective avian population. Moreover, closing the retail live-poultry markets is the fastest control measure in controlling the disease.

The roles of wild birds and domestic birds in the transmission of the H5N1 avian influenza are different and mathematical models have been proposed to include both types of birds (Bourouiba et al. [15], Gourley et al. [16], Lucchetti et al. [8], and Tuncer and Martcheva [17]). It will be very interesting to include both wild birds and domestic birds in modeling the bird-to-human transmission of the H7N9 avian influenza. We leave this for future consideration.
